# Scaling up depot medroxyprogesterone acetate (DMPA): a systematic literature review illustrating the AIDED model

**DOI:** 10.1186/1742-4755-10-39

**Published:** 2013-08-02

**Authors:** Leslie Curry, Lauren Taylor, Sarah Wood Pallas, Emily Cherlin, Rafael Pérez-Escamilla, Elizabeth H Bradley

**Affiliations:** 1 Yale School of Public Health, 60 College Street, New Haven, CT 06520-8034, USA; 2 Yale Global Health Leadership Institute, New Haven, CT, USA

**Keywords:** Scale up, Family health, DMPA, Depo-provera, Low-income settings, Innovation, Global health

## Abstract

**Background:**

Use of depot medroxyprogesterone acetate (DMPA), often known by the brand name Depo-Provera, has increased globally, particularly in multiple low- and middle-income countries (LMICs). As a reproductive health technology that has scaled up in diverse contexts, DMPA is an exemplar product innovation with which to illustrate the utility of the AIDED model for scaling up family health innovations.

**Methods:**

We conducted a systematic review of the enabling factors and barriers to scaling up DMPA use in LMICs. We searched 11 electronic databases for academic literature published through January 2013 (n = 284 articles), and grey literature from major health organizations. We applied exclusion criteria to identify relevant articles from peer-reviewed (n = 10) and grey literature (n = 9), extracting data on scale up of DMPA in 13 countries. We then mapped the resulting factors to the five AIDED model components: ASSESS, INNOVATE, DEVELOP, ENGAGE, and DEVOLVE.

**Results:**

The final sample of sources included studies representing variation in geographies and methodologies. We identified 15 enabling factors and 10 barriers to dissemination, diffusion, scale up, and/or sustainability of DMPA use. The greatest number of factors were mapped to the ASSESS, DEVELOP, and ENGAGE components.

**Conclusions:**

Findings offer early empirical support for the AIDED model, and provide insights into scale up of DMPA that may be relevant for other family planning product innovations.

## Background

**Figure 1:**
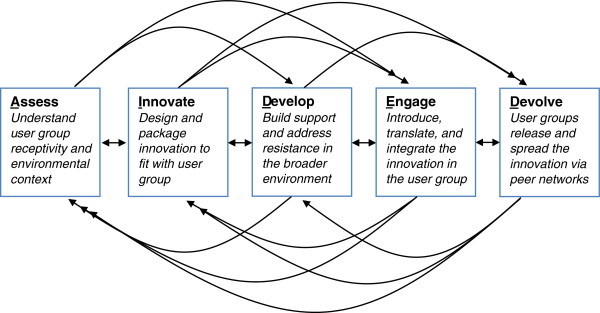
**Schematic of the AIDED model for scaling up family health innovations.** Legend: The figure presents the five non-linear, interrelated actions of the AIDED model: 1) assess the landscape, 2) innovate to fit user receptivity, 3) develop support, 4) engage user groups, and 5) devolve efforts for spreading innovation. The model suggests that successful scale up occurs within a complex adaptive system, characterized by interdependent parts, multiple feedback loops, and several potential paths to achieve intended outcomes. Source: Bradley et al. [9]. Copyright is held by the authors under the Creative Commons License and permission is granted for reproduction in this manuscript.

A puzzle in reproductive health, and public health generally, has long been why innovative products and programs spread widely and rapidly in some contexts but fail to spread in others. Multiple models of spread focus on active dissemination of an innovation, providing recommendations for how to facilitate or accelerate take up [1-5], while other models have described the process of passive diffusion of innovations [6-8]. In work described elsewhere [9], our team identified characteristics associated with successful innovation spread – encompassing both active dissemination and passive diffusion – and developed a new model to capture these dynamics in the domain of family health. The resulting nonlinear, complex adaptive system model, called AIDED (Figure 1), was designed for flexible application across diverse innovation types, including products, behaviors, organizational structures, and delivery systems. The AIDED model thus differs from prior models of reproductive health intervention scale up in that it applies to a broader range of innovations and incorporates both active dissemination and passive diffusion processes, fully recognizing the complex adaptive system nature of scaling up processes.

The description of the AIDED model [9] has consisted of a synthesis of findings across a range of innovation types and may be of use to providers and policymakers interested in family health innovations broadly. However, the current descriptions may be of limited use to those interested in ‘products’ such as injectable contraceptives. This paper provides an in-depth illustration of the AIDED model using results from a systematic review of the academic and grey literature about dissemination, diffusion, scale up, and sustainability of depot medroxyprogesterone acetate (DMPA). DMPA, often known by the brand name Depo-Provera, is a long-acting contraceptive administered by intramuscular injection that is an effective, convenient, reversible, and increasingly popular family planning method [10-14]. Recognition of these benefits, accompanied by approval by the U.S. Food and Drug Administration in 1992, catalyzed a global doubling of injectable contraceptive use between 1995 and 2005, a trend that was particularly prevalent among low-income countries [15]. DMPA scale up has been described in several countries including Bangladesh [16], Uganda [17], Ghana [15], Vietnam [18,19], Taiwan [20], Afghanistan [21], Malawi [22], India [23] and Zimbabwe (Rhodesia) [24]. As a well-documented reproductive health technology that has scaled up in diverse contexts, DMPA offers an exemplar “product-type” innovation in the domain of family health for illustrating the AIDED model’s usefulness. We selected DMPA rather than other injectable contraceptives on account of its longer duration, which is a distinguishing feature that is advantageous in settings where access to health care is difficult; DMPA is also the most prevalent injectable contraceptive used globally [15,25].

Previous literature about scaling up DMPA has tended to focus on the distribution channel, such as whether clinic-based or community-based distribution is used. Numerous findings related to the safety and efficacy of community-based distribution (CBD) of DMPA have been published [10-12,14,23,26-29] and recently synthesized [11] and toolkits to aid policymakers and practitioners in scaling up community-based access to injectables have also been produced [30-33]. By contrast, this paper seeks to synthesize evidence about the enabling factors and barriers to scale up of DMPA *as a product*, rather than scale up of a particular DMPA distribution channel. Our focus is therefore on cases in which DMPA use has spread from a smaller number to a larger number of user groups, regardless of whether that spread occurred via clinic-based or community-based approaches. In this paper we map the evidence about DMPA scale up to the AIDED model, summarizing relevant peer-reviewed and grey literature to illustrate the model with a specific product innovation, and identify lessons for scaling up of DMPA and other contraceptive technologies in low- and middle-income countries.

### The AIDED model

The AIDED model includes 5 non-linear, interrelated components: 1) assess the landscape, 2) innovate to fit user receptivity, 3) develop support, 4) engage user groups, and 5) devolve efforts for spreading innovation [9]. Each component captures a set of processes; this action-based approach distinguishes the AIDED model from other scale-up frameworks that focus on actors or context [2-4,6,34]. The model suggests that successful scale up occurs within a complex adaptive system, characterized by interdependent parts, multiple feedback loops, and several potential paths to achieve intended outcomes. The AIDED model’s nonlinear nature is another distinctive feature relative to existing approaches to scale-up strategy design [1,35]. Importantly, the AIDED model was developed with groups (e.g., organization or community) as its unit of analysis and mechanism of spread, in contrast to prior scale-up studies of individual behavior change [36-38].

The ASSESS component refers to assessment of the broad landscape within a potential user group , including the needs and wants of the user community, its absorptive capacity, and the political, economic, legal/regulatory, technological and social conditions within its internal and external environment. The INNOVATE component includes designing, re-designing, and packaging an innovation so that the innovation is acceptable and perceived as advantageous by potential user groups in their specific context or environment. These processes of designing, re-designing, and packaging the innovation are aimed at achieving ‘fit’ between the innovation and the user group. In the DEVELOP component, attention is directed to fostering enabling relationships, environments and networks among partners that can support and facilitate spread of the innovation. Although engagement occurs throughout the process of dissemination and diffusion, the ENGAGE component involves the specific tasks of introducing the innovation from outside the user group to inside the user group, translating the innovation so that user groups can assimilate the new information, and integrating the innovation into the routine practices and social norms of the user group. Finally, the DEVOLVE component involves the initial user groups releasing and spreading the innovation for its re-introduction in new user groups within their peer networks. These user groups and their networks replicate and release the innovation (in adapted and potentially failed forms) in the way they see most appropriate. The AIDED model is both descriptive of common features of successful innovation spread, and prescriptive of processes that should be considered by those wishing to facilitate scale up.

## Methods

We conducted a systematic review of the literature on the scale up and sustainability of DMPA in low- and middle-income countries. We searched 11 electronic databases including MEDLINE, CINAHL, EMBASE, Web of Science (Science Citation Index and Social Science Citation Index), PsycINFO, Global Health, EconLit, CSA Multi-Search (International Bibliography of the Social Sciences, Social Services Abstracts, and Sociological Abstracts). We included any literature published since the earliest date initialed in each database up to the January 2013 search date. The keywords used to search for articles related to DMPA were medroxyprogesterone acetate, injectable medroxyprogesterone, injectable medroxyprogesterone acetate, DMPA, Depo-, Depo Provera, Depo-Provera, Depo-Provera contraceptive, and Depo-SubQ Provera 104. The keywords used to search for articles related to scaling up were replication, scale up, sustainability, diffusion, dissemination, take up, innovation, diffusion of innovation, technology transfer, information dissemination, acculturation, assimilation, fidelity, and uptake. We used two questions to determine if an article fit our study objective of identifying factors associated with DMPA scale up:

(i) Does the paper specifically address factors related to an increase in the number of individual DMPA users *within a given group or community*?

(ii) Does the paper specifically address factors related to the diffusion, dissemination, or scale up of DMPA use *from one geography to another* (e.g., from village to village, or province to province)?

If the answer to either question (i) or question (ii) was affirmative, the paper was included in our sample. We did not set any minimum criteria for the scope or scale of scale up. We included cases in which DMPA was introduced to end user groups for whom DMPA was new, whether such expanded delivery occurred through task shifting (e.g., from clinic-based providers to community-based health workers) or a first-time introduction of DMPA into a country or health system.

**Figure 2:**
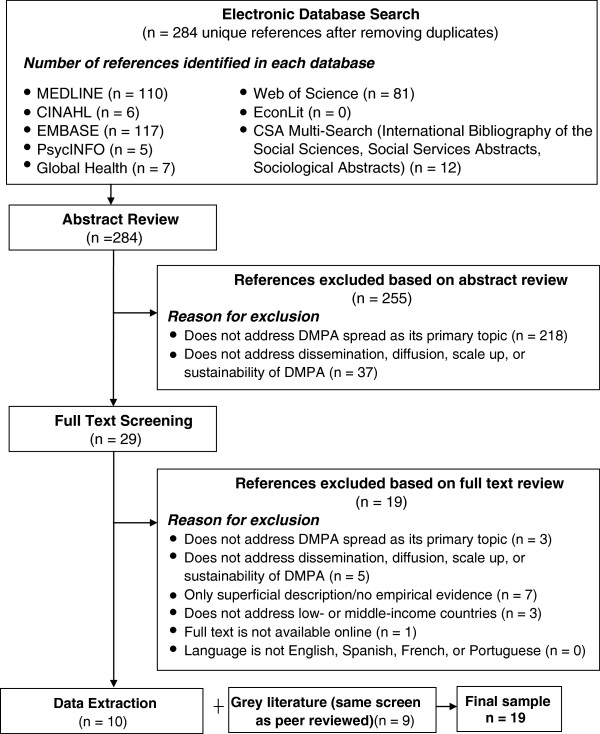
**Peer-reviewed literature review sample selection process.** Legend: The figure summarizes the results of each stage in the search and review process for selecting the sample of peer-reviewed literature. Source: Authors.

The searches yielded an initial sample of 284 unique articles after eliminating duplicates (Figure 2). We screened the abstracts of all articles in this initial sample (n = 284). An article was excluded at the abstract screening stage if it did not address spread of DMPA as an injectable contraceptive as its primary topic (n = 218) or if it did not discuss the scale up or sustainability of DMPA (n = 37). We then reviewed the full text of the articles retained following abstract screening (n = 29). At the full text screening stage, an article was excluded if: (1) it was superficial in its discussion and/or did not provide empirical evidence about the scale up or sustainability of DMPA(n = 7); (2) it did not address scale up or sustainability of DMPA (n = 5), did not address spread of DMPA as an injectable contraceptive as its primary topic (n = 3), did not address low- or middle-income countries (n = 3); or (3) the full text of the article was not available online (n = 1). Following the full text screening, 10 articles were retained for data extraction and analysis.

The grey literature searches targeted the publications/resources databases and websites of the World Health Organization (WHO), the United Nations Children’s Fund (UNICEF), the United Nations Development Programme (UNDP), the United Nations Population Fund (UNFPA), the World Bank, the African Development Bank, the Inter-American Development Bank, and the Asian Development Bank. We also reviewed the project reports published by major international aid organizations [e.g., the United States Agency for International Development (USAID), the Canadian International Development Agency (CIDA), and the Department for International Development (DFID)]. Due to the large volume of hits generated from these web site searches, the titles of all hits were screened first. If a document appeared relevant on the basis of its title, the full text was reviewed using the same exclusion criteria as applied to the academic literature. Finally, we conducted purposeful searches using the same general approach for cases widely recognized as major DMPA implementation initiatives. This process resulted in 9 documents that addressed the scale-up and/or sustainability of DMPA in low- and middle-income countries. Although some characteristics relevant to scale up differ between clinic-based provision and CBD of DMPA, as our focus was on scaling up DMPA as a product innovation rather than scaling up a specific delivery method, we chose to include both delivery methods in this review in order to identify common elements and extract as much information as possible from the limited published literature.

Data extraction from the final sample of academic articles (n = 10) and grey literature documents (n = 9) was conducted independently by two research team members using a pre-established data extraction form. For each article, the data extraction process identified the study design, geographic location, and key findings related to scale up and sustainability of DMPA use. Differences in preliminary data extraction results were harmonized through discussion between the two team members to arrive at a final set of factors influencing the success of DMPA scale up and/or sustainability. Findings were then iteratively grouped into categories of enabling factors and barriers to scale up and/or sustainability, with disagreements resolved through negotiated consensus between the two team members.

After the final list of enabling factors and barriers was established, two team members mapped these scale-up determinants to the five components of the AIDED model (ASSESS, INNOVATE, DEVELOP, ENGAGE, and DEVOLVE). The mapping was conducted by comparing each enabling factor or barrier against the definitions of the five components to determine if it fit into one or more parts of the AIDED model. For example, the enabling factor of “ensuring fit with cultural norms” was mapped to both the ASSESS and INNOVATE components because ensuring fit with cultural norms requires identifying the extant norms (an aspect of assessment) and then tailoring the product to be acceptable within those norms (an aspect of innovation design and packaging). This process preserved the potential for an enabling factor or barrier not to match the definition of any of the five components. Disagreements between the two team members were resolved through negotiated consensus.

## Results

The final sample of 19 sources (10 academic articles and 9 grey literature documents) included studies representing a wide range of geographies and methodologies (Table 1). These studies examined DMPA programs from 13 unique countries; 3 studies included multiple countries. Among the literature from which we extracted data, 5 of the studies used qualitative methods, such as in-depth interviews, focus groups, or observations, 3 sources presented findings from pre/post interventional studies, 3 used mixed methods (in which literature reviews and quantitative analyses were supplemented by key informant interviews), 3 were cross-sectional, 2 were literature reviews or commentaries, and 1 involved time-series modeling. Two of the papers failed to report their methodologies (Table 1).

**Table 1:** Characteristics of final literature sample (n = 19 sources)

	**# of sources**
**Methodology**	
Qualitative interview, focus groups or observations	5
Cross-sectional interviews, questionnaires or chart review	3
Pre-post intervention without comparison group	3
Literature review or commentary	2
Simulation modeling	1
Mixed methods	3
Methods not described	2
**Geographic area**	
Uganda	3
Thailand	2
Afghanistan	1
Ghana	1
India	1
Indonesia	1
Madagascar	1
Malawi	1
Philippines	1
Taiwan	1
Viet Nam	1
Zambia	1
Zimbabwe (Rhodesia)	1
Multiple countries (e.g., literature review)	3
**Administration of DMPA**	
Community-based administration	13
Clinic-based administration	6

The data extraction process identified 15 enabling factors and 10 barriers to dissemination, diffusion, scale up, and/or sustainability of DMPA, all of which were mapped to one or more of the AIDED model components. The ASSESS, DEVELOP, and ENGAGE components of the AIDED model had the largest number of factors and barriers mapped to them (Tables 2 and 3). In the following section, we summarize the factors (or key activities) and barriers identified in the literature as they relate to each component of the AIDED model, and provide illustrative examples for each. We present four general lessons characterizing the scale up of DMPA that may also have relevance for scale up of other family health innovations in low- and middle-income countries.

**Table 2:** Enabling factors for the dissemination, diffusion, scale up, or sustainability of DMPA by AIDED model components

**Enabling factor**	**# sources citing factor**	**AIDED model component(s) to which factor was mapped**
Development of delivery system supports (training of health workers/field motivators, creation of training manuals or checklists, supply chain improvements, recruitment of women, chart tracking)	10	Develop
Tailoring innovation to existing system capacity (community-based distribution systems already in place, women in community health worker roles, other existing program infrastructure (e.g., well-baby clinics, current supply chain flows))	9	Innovate, Devolve
Landscape or stakeholder assessment	6	Assess
Collaboration with stakeholders to identify or create supportive structures in the economic, political and technological spheres	6	Assess, Develop
Use of social networks	5	Devolve
Dialogue with community at early stages	5	Assess, Engage
Effective education through social marketing regarding risks and instructions (including community input)	4	Develop, Engage
Piloting to determine feasibility	3	Assess
Innovation design features (e.g., injectable at 3 month intervals)	3	Innovate
Ensuring ‘fit’ with cultural norms (e.g., allowing women to take injections in strict confidence without being observed)	3	Assess, Innovate
Use of data to improve program performance	3	Engage
Nationalistic messaging (e.g., population control)	2	Develop
Adherence to religious norms (e.g., support of leaders)	1	Innovate, Develop, Engage
Identifying potential sources of resistance, such as from the professional medical community	1	Assess
Creating structures to ensure use of assessment findings through implementation and scale up (e.g., the same individuals that conducted the assessment remained involved throughout the process of scaling)	1	Assess

**Table 3:** Barriers to the dissemination, diffusion, scale up, or sustainability of DMPA by AIDED model components

**Barrier**	**# sources citing factor**	**AIDED model component(s) to which factor was mapped**
Lack of system capacity (e.g., delivery or administrative challenges, lack of equipment, supply chain stock-outs due to mismanagement, staff burden)	5	Innovate, Develop
Rural nature of program areas (e.g., difficulties in maintaining supply chain or human resource levels)	5	Devolve
Inadequate resources for scaled-up activities	4	Devolve
Competing alternatives (e.g., other types of family planning products such as condom, diaphragm, or pill)	3	Develop
Misaligned government policies and priorities (e.g., preference for HIV/AIDS projects, longer acting methods, or provision of family planning by medical personnel)	3	Assess, Develop, Devolve
Data collection challenges (e.g., insufficient contact between front line and supervisors, front line failure to understand tools, follow-up challenges)	3	Develop
Social or cultural norms (e.g., male dominance or power, elder family member objections, general concerns about fidelity and family size)	1	Assess, Innovate, Engage,
Lack of knowledge or awareness (e.g., inadequate counseling or patient education, lack of patient-centered care, limited information sharing)	1	Develop, Engage
Opposition by medical professionals	1	Assess, Engage
Lack of ongoing stakeholder support (e.g., key leaders left after pilot phase)	1	Devolve

### Assess

#### ***Key enabling factors***

In the literature we reviewed, enabling factors that were mapped to the ASSESS component included conducting broad landscape and stakeholder assessments from community to international levels (n = 6), dialogue with the community at early stages to understand cultural and religious norms relevant to contraception and family planning (n = 5), piloting to determine feasibility in the particular context (n = 3), creating structures to ensure use of assessment findings through implementation and scale up (n = 1), and identifying potential sources of resistance (n = 1).

#### ***Barriers***

Three barriers to scale up were mapped to the ASSESS component: misaligned government policies (e.g., favoring provision of contraceptives by medical personnel) (n = 1), opposition by medical professionals (n = 1) and social and cultural norms and dynamics (n = 2). A landscape assessment might have identified and addressed such barriers early in the process, ultimately facilitating scale up.

#### ***Illustrative example***

The process of conducting a comprehensive baseline assessment was described as critical to the introduction of DMPA as part of a package of family planning interventions in Vietnam [18]. In 1994, guided by the Strategic Approach to Contraceptive Introduction sponsored by the World Health Organization [39], the Vietnamese government alongside several international partners began the intervention planning process with a participatory needs assessment, carried out by the Ministry of Health, the National Committee of Population and Family Planning, and the Vietnam Women’s Union. The purpose of the assessment was to determine the suitability and need for contraceptive introduction within a larger initiative to strengthen quality of care in the service delivery system. A dissemination workshop followed in 1995, in which stakeholders reviewed and approved the assessment findings; the pilot intervention began in 1996. Though time intensive, this process served to generate consensus on a dual goal: to improve quality while successfully introducing DMPA to broaden the range of contraceptive choices for women. Individuals who had participated in the strategic assessment were subsequently involved in the design and management of the pilot studies, ensuring continued responsiveness to the issues identified through the assessment. This core team also became the resource team to provide supervision, guidance and mentoring in scale up efforts, as the project grew in scale from 4 to 21 provinces (DMPA is now available in all 64 provinces). In addition to the national level process, tailored, focused assessments were carried out to inform implementation at the local level. Situational analysis was conducted at each of the four pilot sites before implementation; findings were used to improve client flow, logistics and infection control practices at the sites.

### Innovate

#### ***Key enabling factors***

In the literature we reviewed, enabling factors mapped to the INNOVATE component included tailoring the innovation to the existing system capacity, such as infrastructure for well-baby clinics and existing supply chain flows (n = 9), creating innovative design and packaging features (n = 3), ensuring ‘fit’ between design and socio-cultural norms (n = 3), and tailoring innovations to suit current system capacity (n = 9). In general, design features for a product innovation include how the product will be delivered to end users. In the case of DMPA, the ability for a woman to receive DMPA injections every three months without a daily or pericoital regimen, and usually in a private room without others being aware of her contraception use, were critical characteristics of the product that enabled scale up in several low-income contexts. These features figure prominently in the marketing of DMPA and stand in contrast to other types of contraceptive products, such as oral pills or condoms, that are more likely to be observed by or require negotiation with other household members.

#### ***Barriers***

Two barriers identified in the literature mapped to the INNOVATE component: lack of system capacity for DMPA distribution (e.g., delivery or administrative challenges, lack of equipment, supply chain stock-outs due to mismanagement, staff burden) (n = 5) and social and cultural norms and dynamics (n = 1).

#### ***Illustrative examples***

Illustrations of the design, redesign, and packaging of DMPA were highly diverse across the case reports. In one case report from Rhodesia (now Zimbabwe), for instance, the degree of ‘fit’ with cultural and religious norms was defined as a key element. These norms were manifest in a strong taboo against women controlling fertility, with men exercising dominance over women’s reproductive choices out of a desire to control family size and ensure marital fidelity. DMPA’s injectable form allowed ‘fit’ despite these norms. Administered every 3 months, DMPA enabled women to receive the injection in strict confidence, allowing women to navigate the traditions of patriarchal authority without creating friction in their own relationships [24]. The “private acceptors,” as the literature refers to them, were married women who used DMPA without consent or knowledge of husbands. This confidential use was facilitated by mobile well-baby clinics that also supplied contraceptives and an approved system of bookkeeping that allowed private acceptors’ records to be segregated from others to ensure confidentiality. The scale up of DMPA in Zimbabwe (Rhodesia) was substantial; between 1994 and 2006, the proportion of women choosing injectables for contraception rose from 3% to 10% [15].

The importance of the messaging aspect of the innovation’s design was illustrated in the experience of Afghanistan. In addition to detailed information on effectiveness and safety, quotations from the Quran (the holy book of Islam) on the value of birth spacing and breastfeeding were included in the packaging of DMPA. Each quotation was approved by religious leaders known as *mullahs* to allow women to feel that their contraceptive choice was endorsed by the religious structures in the community. Program staff identified the increased social acceptability provided by this kind of packaging as a factor in scale up of DMPA. Overall, the absolute number of women using DMPA doubled and the proportion of women in target groups using DMPA increased from 14% to 40% [21].

### Develop

#### ***Key enabling factors***

In the literature we reviewed, enabling factors mapped to the DEVELOP component included the development of delivery system supports (n = 10), collaboration with stakeholders to identify or create supportive structures in the economic, political and technological spheres (n = 6), effective education through social marketing (n = 4) and nation-building messaging about DMPA’s value (n = 2).

#### ***Barriers***

Five barrier were related to the DEVELOP component, suggesting the importance of directing attention during development efforts to potential impediments. These included lack of system capacity (n = 5), competing alternatives for political or consumer attention (n = 3), misaligned government policies and priorities (n = 3), data collection challenges (n = 3) and lack of knowledge/awareness (n = 1).

#### ***Illustrative examples***

Investment in building and strengthening relationships was common to programs that reported success in scaling up. In Uganda, substantial outreach and advocacy efforts included leadership from the Ministry of Health (MOH) and its non-governmental organization (NGO) allies; these efforts have been fully catalogued in an advocacy guide [32]. In this case, the development of cooperative partnerships between the public and private sector required particular attention to the political climate including key decision makers and influential stakeholders, as well as flexibility to adapt to unforeseen shifts in the political environment. This required MOH and NGO partners to develop clear role definitions for all involved; it was agreed that the public sector would be the primary implementer, with the private sector organizations providing mainly technical assistance. This division of labor facilitated scale up and sustainability as the functions were largely detached from transient funding agendas. Together, both the public and private sectors also undertook “continuous community sensitization” efforts, which aimed at creating awareness and educating the community as to the availability of DMPA [17].

In addition to developing cooperative environments amongst stakeholders, building systems capacity that can support the innovation’s scale up also emerged as an enabling factor. For instance, in Vietnam, where the introduction of DMPA was framed as a quality improvement effort, new management and supervisory practices were introduced before the launch of the intervention, and included management information tools such as a logbook for clients to record side effects and other information. This required substantial investment in training program staff across the system, from the MOH to provinces, individual providers and field motivators. The program adopted a philosophy and practice of supportive supervision (in contrast to inspection and attainment of quotas), which included managers discussing service implementation and problem solving with providers [18].

In India, a USAID-sponsored project introduced in 2003 aimed to scale up availability and access to DMPA in three provinces. The project relied on a private-sector distribution strategy using well-regarded obstetricians and gynecologists to stress the effectiveness and safety of DMPA; however, program managers described the lack of public sector support as being an impediment to success. Specifically, the absence of government endorsement of DMPA in the public sector slowed the pace of growth of the overall market. Not only did the absence of the product from the public health system affect volumes, but as a result some private providers and marketers may have taken a very cautious approach to adopting DMPA themselves [23].

In Zambia, training was essential to making providers more confident about and comfortable with administering DMPA and managing side effects and complications. One of the training approaches involved a popular kit with an innovative system that categorized clients according to lifestyle and then identified the family planning methods that would most likely meet their specific needs. The Central Board of Health recently described this model of profiling clients as a best practice, calling attention to its benefit of grouping clients by needs rather than providing a generic overview of various contraceptive methods [40]. Between 1992 and 2001–2002, injectable use (both Noristerat and Depo-Provera) increased from 0.1% to 4.5%; Depo-Provera was found to be particularly popular and was finally approved for use in the country in 2004 [40].

### Engage

#### ***Key enabling factors***

In the literature we reviewed, enabling factors mapped to the ENGAGE component included: dialogue with community at early stages and throughout implementation (n = 5), effective education through social marketing (n = 4), use of data to improve program performance (n = 3) and compatibility with religious norms (n = 1).

#### ***Barriers***

Three barriers related to the ENGAGE component were identified. They included social and cultural norms and dynamics (n = 1), lack of knowledge and awareness on the part of the community (n = 1), and opposition by medical professionals (n = 1).

#### ***Illustrative examples***

In Afghanistan, local *mullahs* were engaged by program staff to carry the DMPA innovation into the community, where the *mullahs* ultimately grew to play a central role in contraceptive education [21]. Through prolonged and candid discussions, all 37 *mullahs* in the three focal areas accepted the presence of modern birth control (of which DMPA was one option) for the purposes of birth spacing. While their initial role had been to approve DMPA packaging, the *mullahs* soon began preaching about the benefits of injectables at Friday morning prayers. The involvement of these community leaders, all of whom were men, provided additional means by which to inform women of risks and benefits and the injectables’ instructions. The close and visible participation of *mullahs* in contraceptive education was reported as one of several key factors in scale-up success.

At the time of the family planning intervention in Vietnam [18], health care providers had historically been paternalistic in their approach to patient care, particularly in the realm of contraception; this orientation was reinforced by the health care system. The family planning initiative required a major shift in these provider norms toward a patient-centered model of care in which a woman’s autonomous decision regarding contraception was supported. This shift required medical professionals to facilitate patient choice through sharing comprehensive information, and practicing informed consent. These changes were encouraged by supervisors and supported with revised patient education materials for clients stressing voluntary choice. A related feature of the program was aimed at increasing community involvement in health care service planning. Pilot sites were encouraged to seek views of clients and community and to respond to them through action plans and follow up activities; the degree of involvement was monitored and reinforced through quality of care indicators.

Engaging community leaders proved valuable in the introduction of injectables in Ghana and Vietnam [15]. The Navrongo Initiative in Ghana, for example, worked with local opinion leaders and existing men’s and women’s social networks in order to garner community acceptance for modern family planning. Regular community gatherings were held at which influential elders endorsed health care action committees and publicly encouraged open communication around reproductive health. This form of engagement employed by the Navrongo Initiative team was identified as central to increases in women choosing injectable forms of contraception offered by community providers [15]. The role of patient counseling and one-on-one engagement with target users has proven particularly critical in scaling up DMPA. In part, this finding reflects the well-documented side effects of DMPA (e.g., menstrual irregularities including amenorrhea) that many women may find worrisome and which ultimately cause some to discontinue use. Additional benefits of counseling patients extend beyond awareness of side effects. In Vietnam, for instance the DMPA program also resulted in greater attention to clients’ privacy and enhanced recognition of patient informed choice [18].

### Devolve

#### ***Key enabling factors***

In the literature we reviewed, enabling factors mapped to the DEVOLVE component included providing adequate supports such as staff training and clinic space (n = 9) and using peer social networks (n = 5).

#### ***Barriers***

Four barriers at this stage of the process included the rural nature of target program areas (n = 5), inadequate resources for scaled-up activities (n = 4), misaligned government policies and priorities (n = 3), and lack of stakeholder support (n = 1).

#### ***Illustrative examples***

Social networks were reported as a key mechanism in the devolution of DMPA. In Rural Thailand [41], “conversational networks” and interpersonal influence were central to flow of information about family planning and contraceptive choice. Women discussed birth control with neighbors and friends during the course of daily activities (e.g., at the rice mill, the communal well, in the fields). These interactions occurred across age and generation boundaries; however, class and status boundaries were less permeable and information was less likely to be shared across these groups. Furthermore, contraceptive method dominance varied widely between neighboring villages, suggesting that the interlocking networks within a village may make villages less amenable to information from external sources.

In some circumstances, external supports facilitated the devolution process. For instance, in Vietnam [18], substantial attention was directed at supporting scale up of the four pilot programs. Resources from international donor partners and the national government were used to develop a modular toolkit as a guide to adapting and implementing the innovation. Developers of the guide anticipated it would be useful for subsequent sites, yet also expected there would need to be some adaptation to ‘fit’ local contexts. The kit included a comprehensive enumeration of core implementation steps from establishing a task force and conducting situational analysis to identifying appropriate sites through to quality improvement activities.

## Discussion

This systematic review of existing empirical and grey literature identified a limited number of publications of use in understanding the process of successful dissemination, diffusion, scale up, and sustainability of DMPA in low- and middle- income countries. The results illustrate in detail the AIDED model of scale up of family health innovations. Several general lessons are suggested from the findings and may be applicable to the scale up of other family health innovations in low- and middle- income countries.

First, *the design and packaging of innovations should be broadly conceived and iteratively refined in order to ensure ‘fit’ with end user groups*. Attributes of innovation design and packaging are diverse in nature and form; they may include physical properties as well as psychological or emotional aspects of messaging. Taken together, these highly diverse attributes determine the degree to which the innovation will ‘fit’ the needs and wants of the desired user group. Particularly in family planning, where the role of cultural and religious context is paramount [42] and confidentiality around product use can be critical to innovation adoption, deep understanding of the potential user group is central, and is acquired through an iterative process of assessment and engagement. This understanding must be manifest in the design and packaging of the innovation, including the potential need for refinement as scale up unfolds. In the case of the introduction of DMPA in Zimbabwe, understanding cultural norms such as male dominance of sexual relationships and family planning directly informed the design of features to enable women to use DMPA without the knowledge of their husbands [43]. Importantly, design and packaging of an innovation does not happen in isolation or at single, bounded point in time but is rather highly iterative in nature. For instance, as in Afghanistan, the act of engaging opinion leaders (such as religious leaders) can include involving them in packaging and messaging to ensure that qualities of the innovation are compatible with norms.

Second, *innovations should be embedded in existing programs and delivery systems; this requires attention to, and sometimes investment in, both structural and managerial capacity.* The embedding of the innovation within existing systems was commonly described as a core principle, even when investment in developing systems capacity to support the integration of the innovation may be required [17,44,45]. Particularly in cases of CBD of DMPA [46], the development of adequate managerial capacity has proven to be essential for quality and efficiency, for instance in order to ensure injections are being given safely, the supply chain is maintained, and strict confidentiality is preserved. Similarly, structural features of the delivery system can maximize ‘fit’ and therefore increase the likelihood of scale up. In Zimbabwe (Rhodesia), having women (rather than men) deliver injections in non-clinic settings such as well-baby clinics and markets allowed women to use DMPA without the knowledge of others. Systematic assessment of delivery system capacity and harmonization as feasible, particularly where financial and technical capacity is limited, is essential [46].

Third, *anticipating and managing resistance from a variety of constituencies both inside and outside the target user groups is a critical activity throughout the scale-up process.* The constituencies affected by the introduction of the innovation may be highly diverse (e.g., medical professionals in Vietnam and husbands and mothers-in-law in Zimbabwe (Rhodesia)). While the interests and beliefs of some stakeholders are likely to be known in advance of the introduction of an innovation, others may only be identified through the assessment process. Nevertheless, resistance must be anticipated and managed on multiple fronts and throughout the scale-up process. Strategies for addressing resistance may be applied in the innovation design and packaging (e.g., the case of Afghanistan), the development of the environment (e.g., the case of Uganda), and in processes of engagement (e.g., the case of Vietnam). Early and sustained engagement of health officials from all levels can mitigate such resistance; local core teams or ‘resource teams’ are a potentially effective mechanism for engagement [18].

Fourth, *diffusive spread beyond the initial group is enabled by existing networks within the user groups, and may require diverse and continuing support from external entities*. The power and potential of existing peer networks was leveraged in several cases we reviewed [18,41,47]. The literature we reviewed suggested spread of family health innovations through peer networks might not require additional external resources. In one example from Vietnam, the external group that introduced the innovation was very deliberate in investing financial and human resources in the transfer of knowledge activities for scale up beyond the pilot sites. This took the form of a training kit that was intended to provide guidance about essential programmatic elements that could also be adapted for local context. In addition to knowledge transfer activities, external entities may also provide various financial and non-financial supports to strengthen or create new user group networks to facilitate spread [18].

### Limitations

This study had several limitations. First, many of the articles in our sample did not describe all stages of the scale-up process in equivalent levels of detail. As a result, we were unable to disaggregate our analysis in terms of the scope, pace, or extent of scale-up in the cases we examined. Second, the sample included few quantitative studies; thus we were not able to evaluate the statistical associations between each identified factor and scale-up. Finally, as a systematic review of the academic and grey literature, this study does not include data that is unpublished, and therefore may over-represent features of DMPA scale-up that can be measured and described more easily.

## Conclusion

Available empirical and grey literature about DMPA provides support, in varying degrees, for each of the 5 components of the AIDED model. An implication of our study is that rigorous evaluation of DMPA scale-up needs to continue and accelerate in order to support evidence-based programming in this area. An important area for future research would be to analyze the presence of the different AIDED model components according to the scope, pace, and extent of scale-up to determine whether more AIDED characteristics were present in projects that were scaled-up faster, implemented at greater scale, or sustained over a longer period of time. Another area for future research is the application of the AIDED model to the scale-up of different distribution systems for DMPA, specifically clinic-based versus community-based, to determine if particular AIDED model components are differentially represented across these distribution approaches. Future research should also specifically investigate the DEVOLVE component of the AIDED model with respect to DMPA scale-up, for which evidence was particularly limited in the literature we reviewed. There is a need to better understand how social networks facilitate the spread of DMPA use within and across end user groups and to document best practices to facilitate such diffusion. Further research on the concrete strategies by which front-line staff have devolved program management and leadership to the communities for the purposes of sustainable spread is also necessary, as this process is the least often reported in the literature.

Understanding facilitators and barriers to the scale-up of DMPA is useful, given the strong interest in the potential of DMPA in family health and growing evidence of the efficacy and safety of community-based distribution channels. This systematic synthesis of available literature highlights the critical importance of nuanced aspects of ‘product-type’ innovations in family health, as well as the importance of careful appraisal of the context in which the innovation is being introduced. More broadly, our investigation of the DMPA literature upholds the value of the AIDED model in understanding practical scale-up challenges in reproductive health and suggests that it may be a useful framework for public health practitioners seeking evidence-based guidance in scaling up an array of health interventions.

## Competing interests

The authors have no competing interests to declare.

## Authors’ contributions

Conception and design of the study LC, LT, RPE, EB. Analysis and interpretation LC, LT, SWP, EC, EB. Drafting and critically revising the manuscript LC, LT, SWP, EC, RPE, EB. Final approval LT, LC, SWP, EC, RPE, EB. All authors read and approved the final manuscript.
